# Exploring the Connection Between Thyroid Health and Psychiatric Disorders: A Comprehensive Review With a Focus on Schizophrenia and Bipolar Disorder

**DOI:** 10.7759/cureus.102146

**Published:** 2026-01-23

**Authors:** Abdulrahman H Al Qaderi, Abdelaziz A Osman, Haya Manasrah, Mehnaz Z Ali, Nour Al Qaderi

**Affiliations:** 1 Psychiatry, Emirates Health Services (EHS), Dubai, ARE; 2 Psychiatry, Al Amal Psychiatric Hospital, Dubai, ARE; 3 Medicine and Surgery, Emirates Health Services (EHS), Dubai, ARE; 4 Medicine and Surgery, Tawam Hospital, Abu Dhabi, ARE

**Keywords:** autoimmune thyroiditis, bipolar disorder, neuroendocrinology, psychiatric disorders, schizophrenia, thyroid diseases, thyroid function tests, thyroid hormones

## Abstract

The interplay between thyroid dysfunction and major psychiatric disorders, particularly schizophrenia and bipolar disorder, represents a critical area of research with significant implications for diagnosis and management. Thyroid dysfunction manifests as either hyperthyroidism or hypothyroidism, each exerting distinct effects on psychiatric conditions. This bidirectional relationship underscores the need for a comprehensive understanding to enhance clinical outcomes. This comprehensive review explores the underlying mechanisms linking thyroid dysfunction and psychiatric disorders, including neuroendocrine dysregulation, immune-mediated pathways, and the effects of psychiatric medications on thyroid function. Both hypothyroidism and hyperthyroidism have been implicated in exacerbating psychiatric symptoms such as mood instability, cognitive impairment, and treatment resistance. In addition, thyroid autoimmunity, particularly the presence of thyroid antibodies, has been associated with increased rates of depression and anxiety. A comprehensive literature search was conducted using PubMed, Scopus, and Web of Science databases for studies published up to September 2024. Keywords included "thyroid dysfunction," "psychiatric disorders," "schizophrenia," and "bipolar disorder." Both MeSH terms and free-text terms were utilized to maximize relevant study retrieval. Peer-reviewed articles examining the relationship between thyroid health and psychiatric disorders were included, with exclusions for non-English studies and those not involving human subjects. Data extraction was independently performed by three reviewers, capturing study design, sample size, thyroid and psychiatric assessment methods, and main findings. Study quality was assessed using the Newcastle-Ottawa Scale for observational studies and the Cochrane Risk of Bias Tool for randomized controlled trials, evaluating selection bias, study design, confounders, outcome measurements, and statistical analysis. Due to study heterogeneity, a meta-analysis was not feasible. Instead, findings were synthesized narratively, categorizing results by type of thyroid dysfunction (hypothyroidism and hyperthyroidism) and psychiatric condition (schizophrenia and bipolar disorder). The review highlights the prevalence and diagnostic challenges of thyroid dysfunction in psychiatric patients, emphasizing the necessity of integrated care approaches that involve collaboration between mental health and endocrine specialists. Understanding these interconnections may facilitate improved screening, diagnosis, and treatment strategies, ultimately contributing to better patient outcomes. This paper seeks to enhance existing knowledge and propose future research directions aimed at optimizing diagnostic and therapeutic practices for individuals affected by these comorbidities.

## Introduction and background

Psychiatric disorders, especially schizophrenia and bipolar disorder, are among the most severe mental health conditions worldwide. Both conditions significantly affect overall health and quality of life. The connection between psychiatric disorders and physical health is well-established, with increasing evidence showing a bidirectional relationship between mental health and endocrine function, particularly thyroid health [1,2].

The thyroid gland plays a vital role in regulating metabolism by producing hormones like thyroxine (T4) and triiodothyronine (T3), which are essential for growth, development, and physiological stability, including proper brain function. Thyroid disorders, such as hypothyroidism, hyperthyroidism, and the effects of thyroidectomy, have significant impacts on mental health [3,4].

Conversely, psychiatric conditions can influence thyroid health through various mechanisms, including neuroendocrine regulation, inflammatory pathways, and the effects of psychiatric medications [5,6].

Understanding the relationship between psychiatric disorders and thyroid dysfunction is essential for improving patient care, as individuals with schizophrenia or bipolar disorder may also experience thyroid issues that complicate their treatment [2].

The aim of this review is to synthesize current evidence on the relationship between thyroid dysfunction and major psychiatric disorders, with a particular focus on schizophrenia and bipolar disorder. Specifically, this review examines underlying neuroendocrine, immune-mediated, and pharmacological mechanisms linking thyroid health and psychiatric symptoms, highlights clinical implications for screening and management, and identifies key gaps to guide future research.

Background

Schizophrenia and bipolar disorder are chronic psychiatric conditions that profoundly impact the emotional, cognitive, and social functioning of affected individuals. These conditions have a complex etiology involving genetic, neurodevelopmental, and environmental factors, often emerging in late adolescence or early adulthood [5,7,8].

The pathophysiology of schizophrenia involves several neurotransmitter systems, including the dopaminergic, serotonergic, glutamatergic, and GABAergic systems [2]. Dysregulation of these systems contributes to the hallmark symptoms of the disorder. Evidence also points to disruptions in the hypothalamic-pituitary-thyroid (HPT) axis in schizophrenia, suggesting that thyroid health may play a significant role in the development and exacerbation of symptoms [2].

Bipolar disorder, on the other hand, is characterized by mood fluctuations that range from manic or hypomanic episodes to episodes of major depression [9]. Bipolar disorder, similar to schizophrenia, is thought to arise from a combination of genetic factors, neurochemical imbalances, and environmental influences. Moreover, dysregulation in neurotransmitter systems, such as the serotonergic, noradrenergic, and dopaminergic pathways, is implicated in the etiology of bipolar disorder [4,10].

The connection between psychiatric disorders and thyroid dysfunction can be partly explained by the HPT axis, which regulates thyroid hormone levels [3]. Dysregulation of the HPT axis has been observed in patients with both schizophrenia and bipolar disorder [7]. Chronic stress, often experienced by individuals with these psychiatric conditions, can lead to alterations in the HPT axis, contributing to abnormalities in thyroid hormone levels. Additionally, the impact of psychiatric medications, such as antipsychotics and mood stabilizers, on thyroid health further complicates the relationship between these disorders [11].

Inflammation is another mechanism linking thyroid dysfunction with psychiatric disorders. Elevated levels of pro-inflammatory cytokines have been found in both schizophrenia and bipolar disorder, which may influence thyroid function and contribute to the development of thyroid abnormalities [4]. The presence of thyroid antibodies, specifically in autoimmune thyroiditis, has also been associated with increased rates of depression and anxiety, highlighting the bidirectional interaction between the immune system, thyroid health, and psychiatric well-being [10].

## Review

Thyroid disorders and their relationship with psychiatric health

Thyroid Dysfunction in Schizophrenia

Thyroid dysfunction, particularly hypothyroidism and hyperthyroidism, is prevalent among individuals with schizophrenia. The connection between thyroid health and schizophrenia is supported by evidence showing altered levels of thyroid hormones, such as T4 and thyroid-stimulating hormone (TSH), in patients with schizophrenia [10]. Several studies have highlighted that individuals with schizophrenia are at an increased risk of thyroid dysfunction, which may be attributed to both the pathophysiology of the disorder and the side effects of antipsychotic medications [3].

As discussed earlier, the HPT axis plays a crucial role in regulating thyroid hormone levels, and dysregulation of this axis has been observed in schizophrenia [11]. Disruptions in the HPT axis can lead to altered levels of TSH, T4, and T3, which have been associated with both positive and negative symptoms of schizophrenia. Moreover, thyroid hormone abnormalities are thought to contribute to the cognitive deficits and mood disturbances observed in patients with schizophrenia [10].

Thyroid dysfunction in schizophrenia has been linked to the dopaminergic, serotonergic, glutamatergic, and GABAergic neurotransmitter systems [10]. Dysregulation of these neurotransmitter systems, which are involved in mood regulation, cognition, and behavior, may be influenced by altered thyroid hormone levels [12,13].

In addition, a recent meta-analysis found that patients with first-episode psychosis (FEP) had significantly lower TSH levels, while patients with multiple-episode schizophrenia (MES) had elevated TSH levels compared to healthy controls [10]. These findings highlight the importance of routinely monitoring thyroid function in patients with schizophrenia, particularly starting from the onset of psychosis. Furthermore, chronic antipsychotic use has been shown to contribute to the development of subclinical hypothyroidism in patients with schizophrenia [12,13]. This effect is primarily due to the impact of antipsychotics on the HPT axis, where they can interfere with the normal regulation of TSH. Antipsychotic medications, particularly those that increase prolactin levels by blocking dopamine receptors, can suppress the release of TSH, leading to reduced stimulation of the thyroid gland. Over time, this can result in decreased production of thyroid hormones, contributing to subclinical hypothyroidism. Studies have also suggested that the risk of thyroid dysfunction increases with prolonged use of antipsychotics, highlighting the need for regular monitoring of thyroid function in these patients [14,15].

Early research on thyroid supplementation in patients with schizophrenia demonstrated significant improvements in symptoms when high doses of natural thyroid extract were administered [7]. This suggests that thyroid hormones may play a role in modulating schizophrenia symptoms, particularly when standard antipsychotic treatments are inadequate. Autoimmune thyroiditis, characterized by elevated anti-thyroid peroxidase (anti-TPO) antibodies, has also been implicated in psychosis, with several studies reporting increased antibody prevalence among individuals with schizophrenia, suggesting an immune-mediated contribution to disease pathology [9].

Thyroid Dysfunction in Bipolar Disorder

Thyroid dysfunction is prevalent among individuals with bipolar disorder, particularly in those treated with mood stabilizers such as lithium, which is associated with an increased risk of hypothyroidism [6]. Thyroid dysfunction can exacerbate mood symptoms and contribute to treatment resistance by disrupting serotonergic, noradrenergic, and dopaminergic neurotransmitter systems [12]. Hypothyroidism, in particular, has been linked to worsening depressive symptoms, increased frequency and severity of mood episodes, and reduced effectiveness of mood-stabilizing treatments, including lithium.

Notably, lithium itself contributes to thyroid dysfunction, creating a bidirectional clinical challenge in which treatment of bipolar disorder may inadvertently worsen thyroid health and complicate mood stabilization. Inadequately treated thyroid dysfunction has been associated with persistent mood instability, difficulty achieving remission, and an increased risk of rapid cycling, characterized by four or more manic, hypomanic, or depressive episodes per year [6]. These findings underscore the importance of regular thyroid function monitoring and timely intervention as part of a multidisciplinary treatment approach [16-18].

Individuals with bipolar disorder are more likely to have autoimmune thyroiditis than the general population, which suggests that an immune-mediated mechanism may link thyroid dysfunction and bipolar disorder [6]. The presence of thyroid antibodies, such as TPO antibodies, significantly increases the risk of depressive symptoms in patients with bipolar disorder. TPO antibodies are involved in the destruction of thyroid cells, leading to reduced thyroid hormone production and subsequent hypothyroidism. Thyroid hormone deficiency worsens depressive symptoms and contributes to mood instability [10]. TPO antibodies serve as key indicators of thyroid dysfunction and help identify patients at higher risk of mood disturbances. Detecting these antibodies early enables clinicians to actively manage thyroid health, reducing the risk of worsening psychiatric symptoms [4].

Furthermore, studies have shown that using supraphysiologic doses of thyroid hormone can help manage treatment-resistant depression in patients with bipolar disorder. These higher-than-normal doses of thyroid hormone may enhance mood stabilization and reduce depressive symptoms in cases where standard treatments have proven ineffective. The mechanism behind this benefit is thought to involve the regulation of neurotransmitter systems, such as the serotonergic and noradrenergic pathways, which play crucial roles in mood regulation [16-18] . By optimizing thyroid hormone levels beyond the typical replacement range, these supraphysiologic doses may exert a more pronounced effect on mood stabilization. However, careful monitoring is necessary to avoid potential side effects, such as hyperthyroidism, which underscores the importance of individualized treatment planning when considering this approach [12,13].

Acquired Hypothyroidism and Psychiatric Comorbidities

Acquired hypothyroidism has been associated with an increased risk of developing psychiatric comorbidities, including depression and anxiety, in adult patients. This relationship has been observed in patients both with and without levothyroxine (L-T4) treatment [11,14].

The effectiveness of thyroid hormone treatment in alleviating psychiatric comorbidities in patients with acquired hypothyroidism has been a topic of considerable interest [12]. Studies have shown that L-T4 treatment can mitigate depressive symptoms in hypothyroid patients, although the effectiveness may vary based on individual factors such as the severity of thyroid dysfunction and the presence of thyroid antibodies [4].

Structural brain changes, including reduced hippocampal volume and alterations in cortical thickness, have also been reported in patients with hypothyroidism. These changes may contribute to the cognitive impairments and mood disturbances observed in these patients, highlighting the importance of early diagnosis and treatment of thyroid dysfunction to prevent long-term neurological and psychiatric complications.

Impact of thyroidectomy and thyroid health on psychiatric outcomes

Thyroidectomy and Psychiatric Manifestations

The absence of thyroid hormone production following thyroidectomy can lead to a range of psychiatric symptoms, including depression, anxiety, and cognitive dysfunction [8]. The relationship between thyroidectomy and psychiatric health underscores the importance of careful preoperative assessment and postoperative monitoring of patients for psychiatric symptoms [11].

Patients who underwent thyroidectomy had a significant decline in quality of life, with many experiencing symptoms of depression and anxiety within the first year after surgery [8,14]. This finding emphasizes the need for integrated care involving both endocrinologists and mental health professionals to address the potential psychological impact of thyroidectomy. 

Thyroid Dysfunction and Psychosis

Hyperthyroidism has been noted to present with psychosis as the first symptom in some individuals, underscoring the need for careful differential diagnosis in patients presenting with acute psychotic episodes [11]. Psychosis as the initial manifestation of hyperthyroidism can be particularly challenging to recognize, as it may be misattributed to a primary psychiatric disorder rather than an underlying endocrine condition [12-15].

In addition, several clinical features of thyroid dysfunction overlap with those of psychotic and affective disorders, including intense anxiety, agitation, irritability, insomnia, and marked mood fluctuations. These shared symptoms may obscure recognition of an underlying endocrine etiology, particularly in emergency or first-episode psychiatric settings [13]. In rare but critical cases, thyroid storm may present with severe agitation, confusion, or paranoid ideation that can closely mimic acute psychosis; however, this presentation is typically accompanied by systemic manifestations such as hyperthermia, tachycardia, gastrointestinal disturbances, and autonomic instability [13,14]. Awareness of these overlapping and distinguishing features is essential to avoid misdiagnosis and to ensure timely medical evaluation and treatment.

Thyroid hormone replacement therapy has shown promise in mitigating psychotic symptoms in patients with thyroid dysfunction. In patients with hypothyroidism, L-T4 treatment has been reported to alleviate psychotic symptoms and improve overall psychiatric outcomes [16]. 

Thyroid Function and Mood Disorders Following Thyroidectomy

Thyroid hormones play a crucial role in neurodevelopment and neuroplasticity. Hypothyroidism has been linked to impaired neurogenesis, particularly in the hippocampus, a brain region involved in mood regulation and cognitive function. Reduced hippocampal neurogenesis may contribute to the development of depressive symptoms in patients with thyroid dysfunction [17].

Psychiatric disorders as a cause of thyroid dysfunction

Psychiatric disorders, including schizophrenia and bipolar disorder, have been implicated as potential contributors to thyroid dysfunction. This relationship is partly mediated by stress-induced dysregulation of the HPT axis, as described above, which plays a central role in regulating thyroid hormone production and secretion [11]. Chronic stress and the activation of the HPT axis, often observed in individuals with severe psychiatric disorders, can result in alterations in thyroid hormone levels, leading to either hypothyroidism or hyperthyroidism [6].

Mechanisms of HPT axis dysregulation

The HPT axis is influenced by several neurochemical and hormonal factors, including cortisol, which is elevated in response to stress. In patients with psychiatric disorders, chronic activation of the hypothalamic-pituitary-adrenal (HPA) axis leads to increased cortisol levels, which in turn impacts the HPT axis and disrupts normal thyroid function [12]. This dysregulation can lead to decreased secretion of TSH and reduced levels of thyroid hormones [11].

Impact of Psychiatric Medications on Thyroid Health

 Lithium inhibits the release of thyroid hormones by affecting iodide uptake and reducing TPO activity, thereby impairing the production of T3 and T4 [11]. Antipsychotics, particularly first-generation antipsychotics, can increase prolactin levels by blocking dopamine receptors in the hypothalamus. Elevated prolactin levels have been associated with decreased TSH secretion and subsequent hypothyroidism [13,19]. 

Limitations

This review has several limitations that should be acknowledged. As a narrative review, the findings are inherently dependent on the scope and quality of the available literature and may be subject to selection bias despite the use of multiple databases and predefined search terms. Additionally, heterogeneity across included studies in terms of study design, diagnostic criteria, patient populations, and thyroid function assessment methods limited direct comparability and precluded quantitative synthesis. The predominance of observational evidence further restricts causal inference. Finally, potential confounding effects of psychiatric medications, comorbid medical conditions, and illness severity may not have been consistently accounted for across studies. These limitations highlight the need for well-designed longitudinal and mechanistic studies to further clarify the relationship between thyroid dysfunction and psychiatric disorders.

Recommendations for future studies

The relationship between psychiatric disorders, particularly schizophrenia and bipolar disorder, and thyroid dysfunction is gaining increasing interest. Despite growing evidence, there are still several gaps in the current research. To address these gaps, future research should prioritize the following areas: 1) Prospective longitudinal studies: Conduct large-scale, longitudinal studies to determine the causal relationship between thyroid dysfunction and psychiatric disorders. Such studies can determine the direction of these relationships and assess whether early thyroid abnormalities contribute to psychiatric symptoms or vice versa. 2) Mechanistic studies: Investigate the precise mechanisms underlying the bidirectional relationship between thyroid dysfunction and psychiatric disorders. Focus should be placed on understanding how the HPT axis interacts with neurotransmitter systems, such as the dopaminergic and serotonergic systems, and immune responses in patients with schizophrenia and bipolar disorder [11-14]. 3) Standardized guidelines: Develop standardized guidelines for the assessment, monitoring, and management of thyroid function in psychiatric patients that involve both endocrinologists and psychiatrists. 4) Impact of psychiatric medications on pathogenesis:It is critical to further explore the role of specific psychiatric medications, such as atypical antipsychotics and mood stabilizers, in the pathogenesis of thyroid dysfunction.

## Conclusions

The intricate relationship between psychiatric disorders like schizophrenia, bipolar disorder, and thyroid health highlights the importance of an integrated approach to patient care. Thyroid dysfunction can both contribute to and exacerbate psychiatric symptoms, while psychiatric medications and chronic stress can negatively impact thyroid function. Understanding and addressing these connections are crucial for improving patient outcomes. Future research should focus on establishing causal links, understanding underlying mechanisms, and developing standardized care protocols. Collaborative care involving both mental health and endocrine specialists will be key to effectively managing these patients and improving their quality of life.
